# The maintenance of stable yield and high genetic diversity in the agricultural heritage torreya tree system

**DOI:** 10.1186/s12898-019-0256-6

**Published:** 2019-09-18

**Authors:** Jian Zhang, Liangliang Hu, Liang Guo, Weizheng Ren, Lufeng Zhao, Ningjing Wang, Entao Zhang, Jianjun Tang, Xin Chen

**Affiliations:** College of Life Sciences, Zhejiang University, Zijingang Campus, No. 866 Yuhangtang Road, Hangzhou, 310058 China

**Keywords:** Torreya tree system, Planting pattern, Seed yield, Genetic diversity, Gene flow, Farmer activity

## Abstract

**Background:**

Understanding how traditional agriculture systems have been maintained would help design sustainable agriculture. In this study, we examined how farmers have used two types of local trees (*Torreya grandis*) for stable yield and maintaining genetic diversity in the “globally important agricultural heritage torreya tree system”. The two type of torreya trees are grafted torreya (GT) tree and non-grafted-torreya (NGT) tree. The GT tree has only female and was used to produced seed yields. The NGT tree has both male and female and was used to support GT tree by providing pollens and rootstocks. We first tested the ratio of GT tree to NGT tree, their age groups, ratio of female trees (including GT and NGT trees) to male, and the flowering period of GT and NGT trees. We then tested seed yields and genetic diversity of GT and NGT trees. We further tested gene flow among NGT trees, and the relationship of gene flow with exchange rates of pollens and seeds.

**Results:**

GT and NGT trees (male and female) were planted in a mosaic pattern with a ratio of 4:1 (GT:NGT). In this planting pattern, one NGT male trees provided pollen for 20 female trees of GT and NGT. The trees were classified into four age groups (I = 100–400 years old; II = 400–700 years old; III = 700–1000 years old; and IV = 1000–1300 years old) based on basal diameter. The entire flowering period was longer for NGT trees than for GT trees that ensured GT trees (which lack of males) being exposed to pollens. GT tree had high and stable seed yield that increased with age groups. High genetic diversity has been maintained in both rootstocks of the GT trees and NGT trees. There was a strong gene flow among NGT trees, which positive correlated with the exchange rates of pollens and seeds.

**Conclusions:**

Our results suggest that farmers obtain stable seed yields, and maintain high genetic diversity by ingeniously using the local GT tree as yield producer and NGT tree as supporter. These GT and NGT trees together ensure sustainable torreya production.

## Background

Traditional agricultural systems (e.g., terrace, poly-culture, and agroforestry systems) have been created by local farmers based on local biological resources and environment [1–6]. These systems contain not only rich agrobiodiversity but also contain indigenous wisdom and local techniques [1, 5]. Recognizing the ecological legacy in these traditional agricultural systems and examining how the traditional farmers use local resources to develop their agricultural systems could help us develop more sustainable agricultural systems [1, 7].

The ancient Chinese torreya tree system, which is located in Zhejiang Province of southern China (29°25′–29°47′, 120°17′–120°38′; Additional file 1: Fig. S1), is one of these traditional agricultural systems that has been continued by local farmers for more than 2000 years [8]. In 2013, this ancient torreya tree system in this part of China was listed as one of the “Globally Important Agricultural Heritage Systems (GIAHS)” by the Food and Agriculture Organization, the United Nation Development Program, and the Global Environment Facility (http://www.fao.org/giahs/giahs-home/en/). The ancient torreya tree system contains a species *Torreya grandis,* which is regarded as a unique lineage in the phylogeny of Taxacea [9] and has been exploited for long time in China [8]. The system has about 1.05 × 10^5^ ancient Chinese torreya trees (*T. grandis*), among which 7.2 × 10^4^ are more than 100 years old, and thousands of them are more than 1000 years old. The diverse age groups of torreya trees form a spectacular landscape merged with the traditional villages, abundant streams, and mountains [8].

In the ancient torreya tree system, seeds of *T. grandis* are the main harvested products. Because of the high contents of proteins, vitamin *A* and unsaturated fatty acids, the seeds are used as a nutritious food, and are also used in medical practice [10, 11]. To obtain and maintain high yield and good quality of the torreya seeds, local farmers used grafting method to maintain two types of torreya trees. Grafting that joins the root system (rootstock) of one plant to the shoot (scion) of another is an ancient agricultural method to clonally propagate and continue desirable genotypes [12, 13]. By using this grafted technique, local farmers linked the shoot (scion) of a desirable cultivar (*T. grandis* cv. *merrillii*), which was selected based on yield and quality of the seeds by local farmers, to the root system (rootstock) of the wild *T. grandis*. A grafted cultivar *T. grandis* cv. *merrillii*, which is hereafter referred to as the grafted torreya or GT, was developed and passed down for generations. The GT tree is propagated by grafting because the *T. grandis* line used to generate this desirable type produces only female trees. Rather than being grafted, the wild *T. grandis* that is hereafter referred to as non-grafted torreya or NGT has both male and female trees [8].

Local farmers planted GT tree and NGT tree (both female and male) together in the mountain area forming a unique complexity population system. Every year, GT tree in this ancient system produces a large number of high-quality seeds that are important for the local economy, while the seeds produced by NGT tree are used to produce the saplings using as rootstocks for GT propagation [14, 15]. Despite the substantial economic and cultural value of this ancient system, its biological and ecological characteristics remain largely unknown.

We hypothesize that the planting pattern of GT tree to NGT trees by local farmers and the practices of using these two types of torreya trees in this ancient system are important in maintaining seed yield and the genetic diversity. To understand whether the structure of torreya tree population affects the stability of seed yield of GT tree, we examine the ratio of GT tree to NGT trees, age groups of GT and NGT trees, ratio of female trees (including GT and NGT trees) to male, and the flowering period of GT and NGT trees. To understand how genetic diversity of torreya tree is maintained, we test genetic diversity by assessing cpDNA and EST-microsatellite loci, and compare GT and NGT trees across six sites and across age groups. We also test the genetic diversity in the age groups of GT rootstocks and gene flow among the NGT trees in the area. We further examine whether the gene flow correlate with exchange rates of pollens and seeds conducted by local farmers among the village in the study area.

## Results

### The planting pattern of torreya trees

**Fig. 1:**
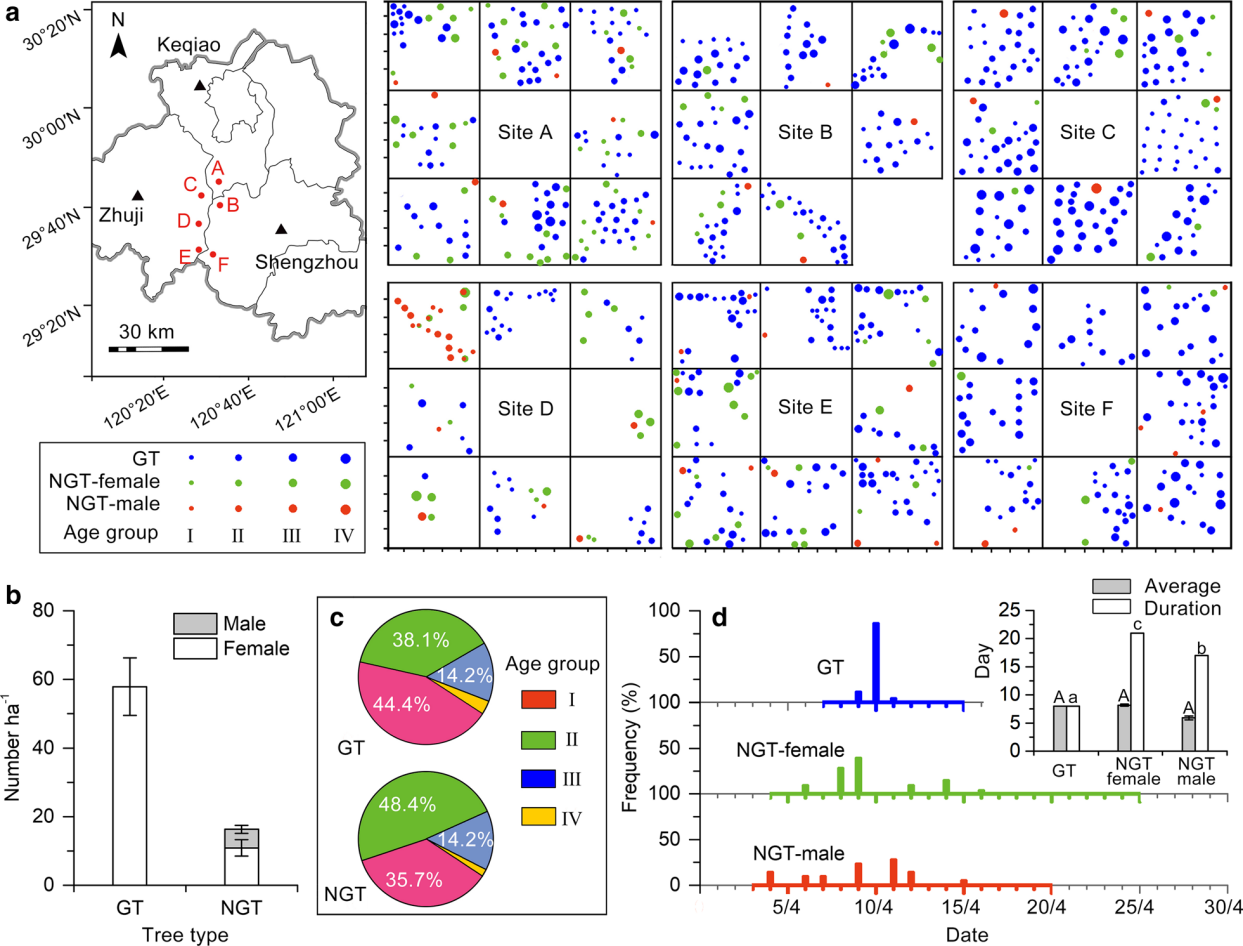
The spatial distribution and characteristics of the two types of torreya trees in the ancient torreya population. **a** Spatial distribution of grafted torreya (GT) and non-grafted torreya (NGT trees). The panel on the left indicates the locations of the six sites (A, B, C, D, E, and F) in the ancient torreya tree system. The panel on the right indicates the distribution of torreya trees in the seven to eight plots at each site; blue, red, and green dots indicate individual GT, NGT male, and NGT female trees, respectively, and the dot size indicates the age group (see Additional file 1: Table S1). Tree age ranged from 100–400, 400–700, 700–1000, and 1000–1300 years in group I, II, II, and IV, respectively. **b** The abundance of GT and NGT trees, and the ratio of female to male NGT trees. Values are mean ± SE. **c** The percentage of GT and NGT trees in each age group. **d** Flowering period of GT and NGT-trees. Blue, green, and red lines along the X axis indicate the duration of flowering, and bars indicate the percentage of GT trees, NGT female trees, and NGT male trees with 30 to 100% of flowers open. The insert indicates the average number of flowering days (from initial flowering to flowering completed) of individual GT, NGT female, and NGT male trees; the insert also indicates duration of flowering per plot (from the date when the first tree in a plot began to flower to the date when the last tree in the same plot completed flowering) for GT, NGT female, and NGT male trees. Values are mean ± SE. *GT* grafted torreya, *NGT* non-grafted torreya

In the torreya tree system, we found that GT and NGT trees were distributed in a mosaic pattern in the GIAHS area (Fig. 1a). GT trees were significantly more abundant than NGT trees (t_5_ = 3.937, P = 0.011), i.e., G T and NGT trees represented 80% and 20% of the population, respectively (Fig. 1b). The sex ratio (female: male) was 20:1 among the whole ancient torreya population (Fig. 1b), indicating that one NGT male tree supporting 20 female trees of GT and NGT trees.

Based on basal diameter, the trees were classified into four age groups: I = 100–400 years old; II = 400–700 years old; III = 700–1000 years old; and IV = 1000–1300 years old (Additional file 1: Table S1). Substantial percentages (from 38.7 to 48.4%) of GT and NGT trees were in group I or group II, intermediate percentages (14.2% in both cases) were in group III, and low percentages (3 to 4%) were in group IV (Fig. 1c).

The entire flowering period was longer for NGT trees than for GT trees (Fig. 1d). Although the number the flowering days per individual tree (flowering initiated to completed) was not longer for NGT trees than for GT trees (F_2,24_ = 1.369, P = 0.284; Fig. 1d, insert), the duration of flowering in a plot was longer for NGT trees than for GT trees (F_2,24_ = 273.520, P = 0.0001; Fig. 1d, insert). The longer duration of flowering for NGT male trees than for GT trees in a plot helps ensure that GT trees (which lack of males) are exposed to pollen.

**Fig. 2:**
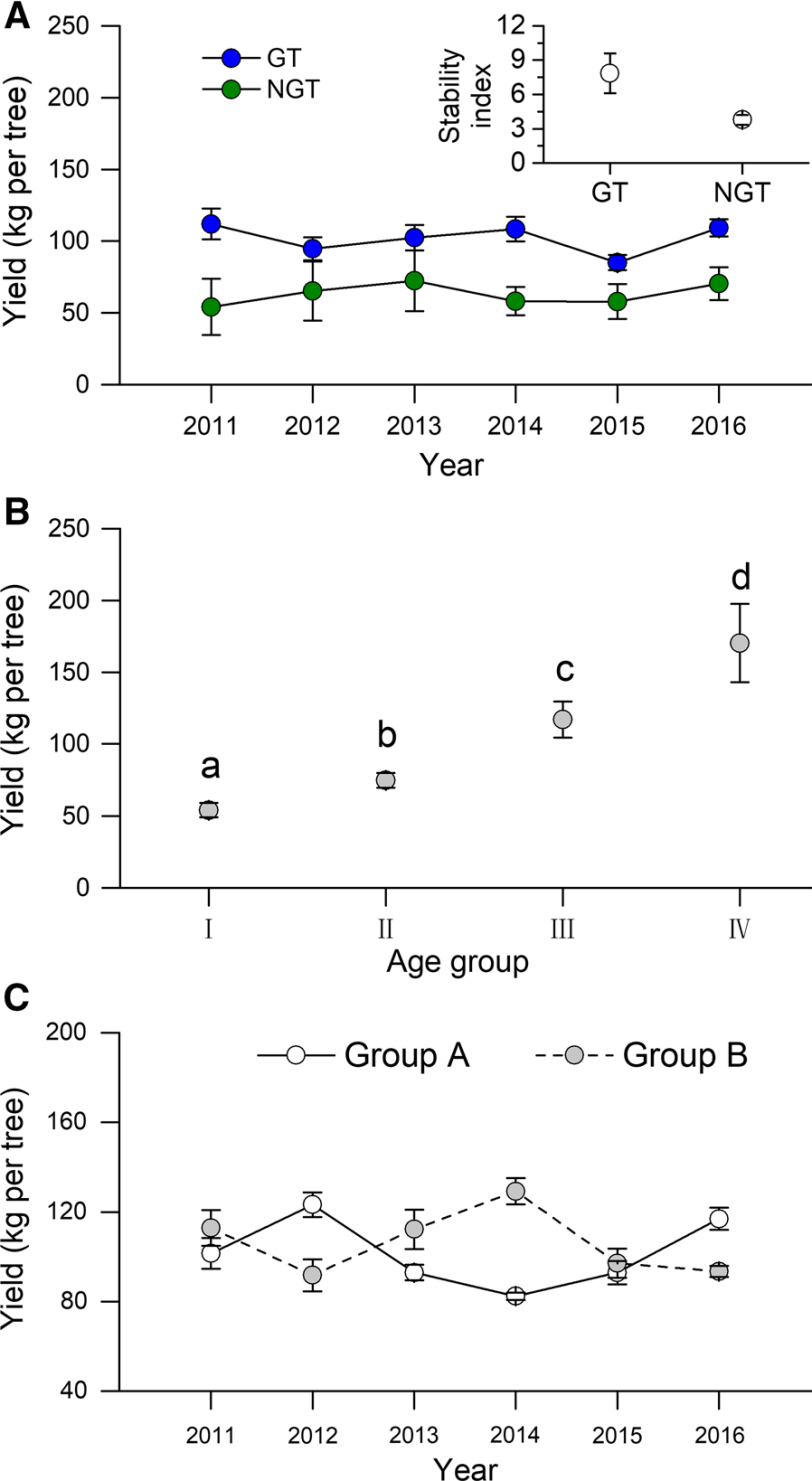
Seed yield in the ancient torreya population as affected by year, tree age, and individual tree. **A** Seed yield per tree and stability of yield from 2011 to 2016. **B** Seed yield per tree for the four age groups of GT trees. Age groups are defined in Additional file 1: Table S1. **C** Variation in the yield of individual GT trees of age group III. The trees were divided into two groups (A and B) based on the temporal pattern of yield. The plot shows that the production of seeds was asynchronous for group A and B. Values are mean ± SE; n = 17 for group A and 28 for group B. *GT* grafted torreya, *NGT* non-grafted torreya

### The yield in torreya tree system

The seed yield and stability index (S) were significantly higher for GT trees than for NGT trees (Fig. 2A; for seed yield, F_1,46_ = 17.842, P = 0.0001; for S, t_6_ = 2.4108, P = 0.049). Seed yield significantly differed among the age groups of GT trees (F_3,12_ = 105.3; P = 0.002; Fig. 2B) and increased with age group. We also measured seed yield of each individual GT tree of age group III from site C. The trees seemed to exhibit two temporal patterns of yield, and we assigned each tree to group A or B based on the patterns. The yields for group A and B confirmed that the variation in yield was temporally asynchronous (Fig. 2C; r = − 0.8267, *t *= − 2.94, P = 0.04).

### The phylogenetic relationship and genetic diversity in torreya trees

**Fig. 3:**
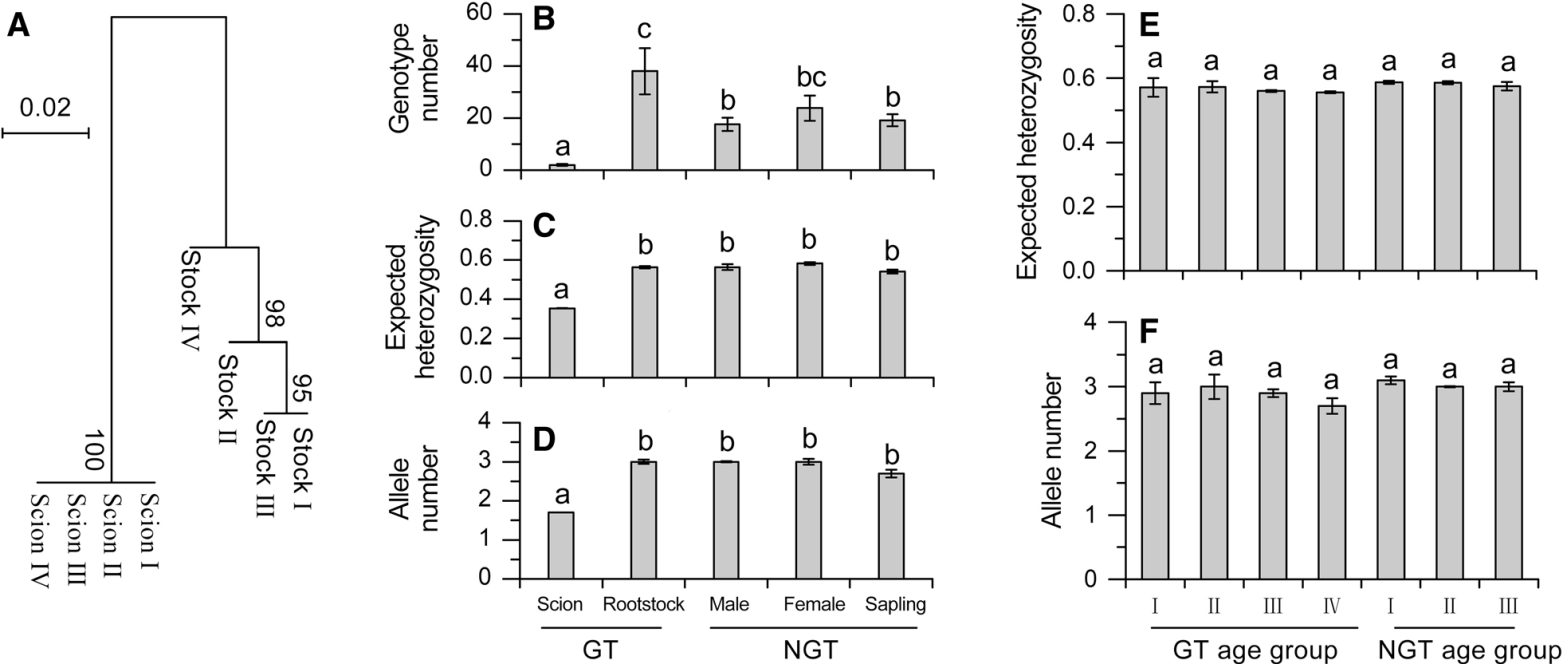
Genetic diversity in the ancient torreya tree system. **A** Neighbor joining tree of different age groups in GT trees based on 20 microsatellite loci. **B**–**D** Genotypes, expected heterozygosity and allele numbers in GT and NGT trees based on 20 microsatellite loci. **E**, **F** Expected heterozygosity and allele numbers in the age groups of GT rootstocks and NGT trees based on 20 microsatellite loci. Values are mean ± SE. *GT* grafted torreya, *NGT* non-grafted torreya

By using *Torreya taxifolia* as an outgroup, a phylogenetic tree based on cpDNA revealed all the scions of GT trees were belong to one identical haplotype, while rootstocks of GT trees, and NGT (male and female trees, and saplings) trees were clustered into a group (Additional file 1: Fig. S2). A neighbor joining tree based on genetic distances obtained from microsatellite analysis also showed that all the scions of GT trees in different age groups were genetically identical, and were different from rootstocks (Fig. 3A).

Microsatellite analysis showed that genotypes based on 20 loci was significantly lower in GT scions than in GT rootstocks or in NGT trees (male, female and sapling) (Fig. 3B, F_4,27_ = 7.03; P = 0.0006). Microsatellite analysis also showed that genetic diversity was lower in GT scions than in GT rootstocks or in NGT trees (male and female) (Additional file 1: Table S2; Fig. 3C, D, for Ne, F_4,27_ = 5.36; P = 0.031; for He F_4,27_ = 1.36; P = 0.023). We also analyzed the genetic diversity of NGT trees and the rootstocks of GT trees at different age groups by using the microsatellite data. In NGT trees or GT rootstocks, genetic diversity was not lower in younger than in older age groups (Additional file 1: Table S3; Fig. 3E, F, for Ne, F_3,4_ = 0.18, P = 0.241; for He F_3,4_ = 0.36; P = 0.223).

The tests of sign, Wilcoxon, and mode-shift showed that both NGT trees and GT rootstocks did not suffer a genetic bottleneck and maintained a considerable effective population size (Additional file 1: Tables S4, S5).

### Source of GT rootstocks

**Fig. 4:**
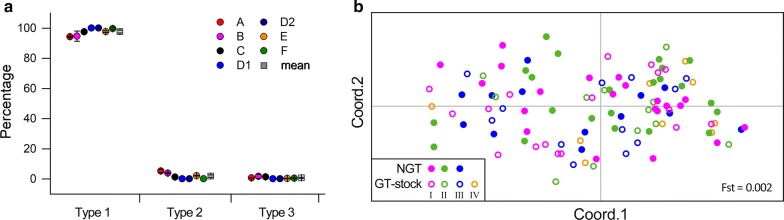
Source of GT rootstocks. **a** The percentage of recent GT rootstocks from NGT saplings grown by farmers. **b** PCoA based on genetic distance among the age groups of GT rootstocks and NGT trees. No genetic pattern among age groups of rootstocks and NGT trees indirectly indicated that NGT saplings have been the historical source of GT rootstocks. Values are mean ± SE. *GT* grafted torreya, *NGT* non-grafted torreya

According to our farmer survey, most recent GT rootstocks were from NGT saplings grown by farmers within the GIAHS area (Fig. 4a). PCoA based on genetic distance also showed that no genetic patterns were found among the age groups of GT rootstocks and NGT trees (Fst = 0.007, Fig. 4b), indirectly indicating the source of rootstocks.

### Gene flow and farmer activities

**Fig. 5:**
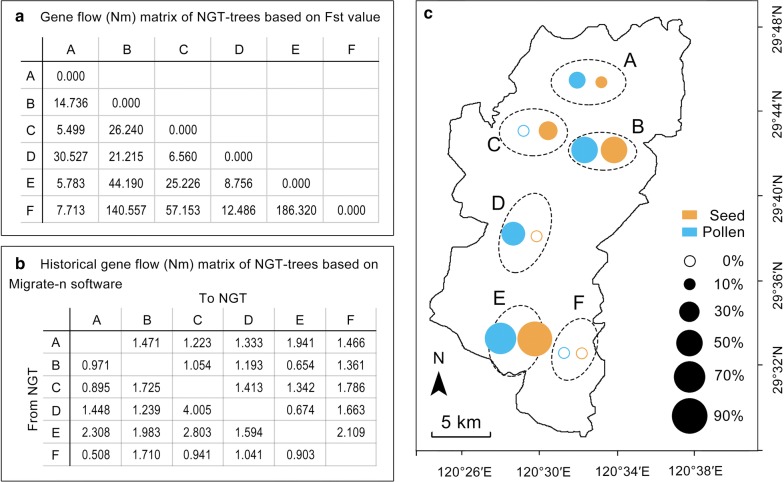
Gene flow and farmer activities. **a** Gene flow based on Wright [28] among NGT trees at the six sites. **b** Historical gene flow based on the coalescent theory among NGT trees at the sites. **c** Flow of pollen and seeds of NGT trees driven by local farmer activities. In **a**–**c**: A, B, C, D, E, and F are the sample sites. *GT* grafted torreya, *NGT* non-grafted torreya

We estimated gene flow among NGT trees at the six sites. The island model based on [16] indicated a strong gene flow among NGT trees at the six sites (Nm > 1) (Fig. 5a). Historical gene flow based on the coalescent theory also indicated a strong gene flow (Nm > 1) among NGT trees at the sites (Fig. 5b).

Our farmer survey showed that local farmers often exchanged pollen or obtained pollen from neighboring villages to ensure high yields of GT seeds (Fig. 5c) because male trees were not evenly distributing in the area (Fig. 1a). Many farmers in the GIAHS area also had exchanged NGT seeds in order to produce saplings when they propagated GT trees via grafting (Fig. 5c).

Mantel test showed that a general correlation pattern was found between pollen and seed exchange rates and gene flow (Table 1). Overall, seed and pollen exchange was moderately correlated with contemporary gene flow (r = 0.408, P = 0.076) and historical gene flow (r = 0.451, P = 0.076). Seed exchange was also moderately correlated with contemporary gene flow (r = 0.404, P = 0.082), and pollen exchange was significantly correlated with historical gene flow (r = 0.664, P = 0.011). The result indicated that farmer activity contributed substantially to the genetic shaping of Torreya population.

**Table 1:** Mantel test between farmer germplasm exchange and gene flow of NGT among the study area

	Contemporary Nm	P value	Historical Nm	P value
Seed and pollen exchange	0.408	0.076	0.451	0.076
Seed exchange	0.404	0.081	0.148	0.254
Pollen exchange	0.295	0.125	0.664	0.011

Nm indicates the effective individual numbers of migration, in which N is the effective population size, m is the migration rate, and Fst is a fixation index (indicating genetic differentiation among populations)

## Discussion

**Fig. 6:**
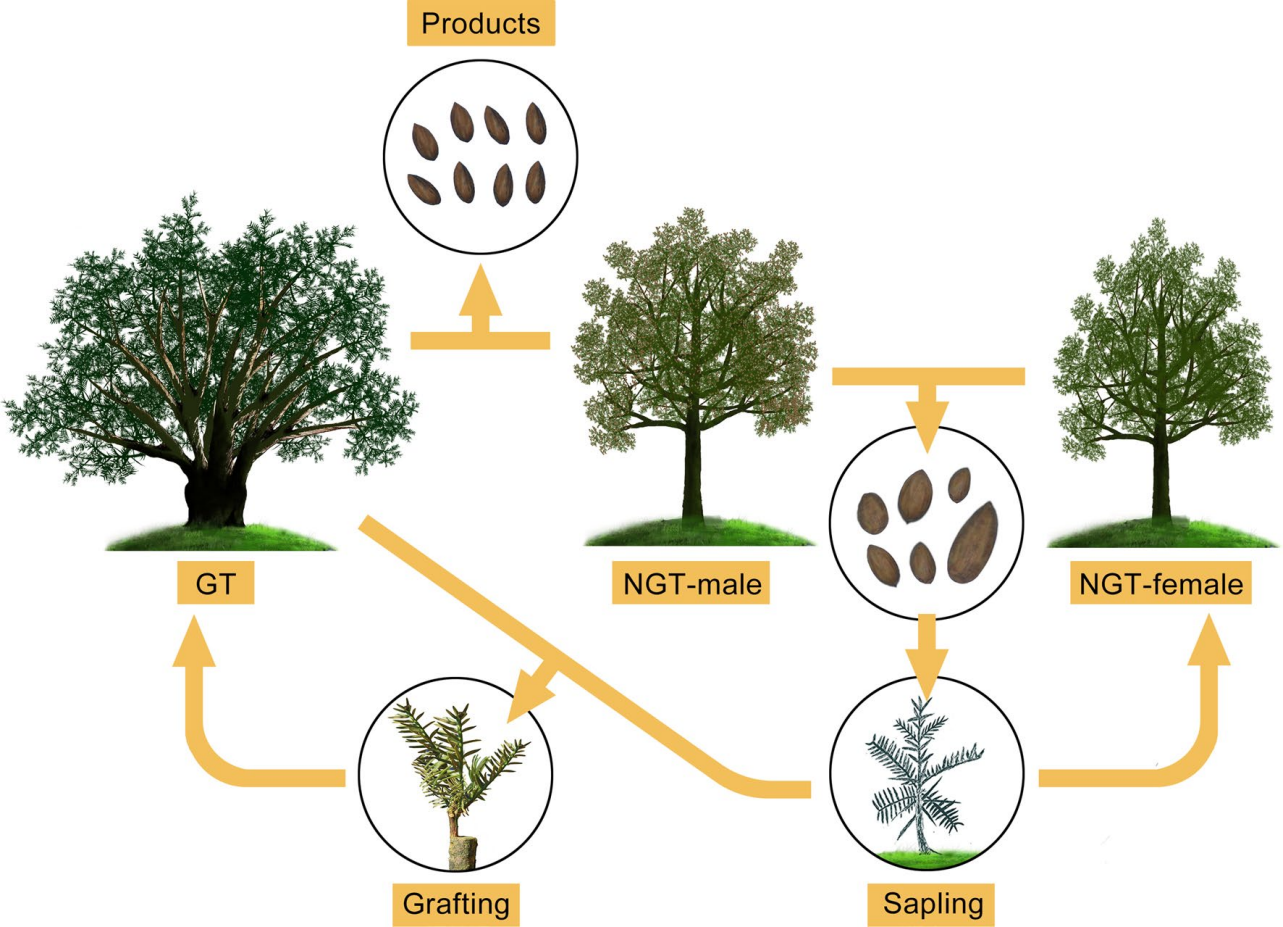
Diagram describing how the grafted torreya (GT) trees and non-grafted torreya (NGT) trees work in the Globally Important Agricultural Heritage System of Chinese torreya. The desirable genotype (grafted torreya, GT), which has genetically uniform scions and genetically diverse rootstock from wild-type torreya trees, is preserved and clonally propagated via grafting. A small number of non-grafted (NGT) trees, both male and female, helps GT trees produce good quality seeds and maintains the genetic diversity of the entire torreya tree system. The NGT male trees provide GT trees with pollen, and the NGT female trees produce seeds that are used to grow saplings that are the main source of GT rootstocks

Our study described how local farmers have maintained the sustainability of the ancient torreya tree system by ingeniously using the two types of torreya trees (GT and NGT trees) (Fig. 6). Results based on cpDNA and microsatellite indicated that all scions of GT trees (from different age groups) were belong to one identical genotype (Additional file 1: Fig. S2, Fig. 3A), while all the rootstocks were genetically similar to NGT trees (Additional file 1: Fig. S2), suggesting that the scions of GT trees have been preserved for thousands of years and NGT trees have been used as the rootstock of GT trees in this area.

Grafting by local farmers plays important role in the maintenance of the torreya tree system. In the torreya tree system, farmers conserved desirable genotype of scion by grafting the scion onto a rootstock from NGT tree that resulted in a GT tree. Farmers planted the GT and NGT trees in a mosaic pattern (Fig. 1a), which GT and NGT trees represented 80% and 20% of the population, respectively (Fig. 1b). The age groups of GT and NGT trees also distributed in a mosaic pattern (Fig. 1a). This complexity of planting pattern resulted in a sustainable valuable seed production in the torreya tree system (Fig. 2). On one hand, seed yield of each GT tree was significantly higher than that of NGT tree, suggesting that high proportion of GT trees resulted in high total seed yield. On the other hand, seed yield of GT tree has a high yield stability index and seed yield of GT tree increased with age group (Fig. 2A, B), implying that high percentage of GT trees could help to maintain stable seed yield of the whole area. The coexistence of age groups in GT trees may also contribute to seed yield stability. In site C, seed yield of each individual GT tree of age group III varied in a pattern of temporally asynchronous (Fig. 2C), such an asynchronicity could offset the fluctuations among individual trees and contribute to the stability of yield at the whole site.

Local farmers also planted small ratio of male trees to female trees (including GT and female NGT trees) in the torreya tree system for high and sustainable yield (Fig. 1b). The torreya tree is dioecious plant, which sex ratio is often an important factor affecting reproduction and fruit or seed yield [17]. In natural ecosystems, the sex ratio of dioecious plants is often about 1:1 (male:female), and this ratio can be shifted by environment [18, 19]. In agricultural systems, the sex ratio of fruit trees is usually determined by the farmers [20]. In the torreya tree system, local farmers planted small numbers of NGT-male trees and NGT-female trees together with GT trees because there was no male trees in GT tree population. The ratio of male trees to female trees (including GT and female NGT trees) was around 1:20, which one NGT-male trees provided pollens for 20 female trees of GT and NGT in the whole system. Because male trees were not evenly distributing in the area (Fig. 1a), local farmers shared their pollens by exchanging pollen or obtaining pollen from neighboring villages to ensure all the female trees pollination. Compared to GT trees, the NGT-male trees had longer flowering period than that can also ensure pollination of GT trees (Fig. 1d).

Although seed yield produced by NGT trees was lower and not used as important food [8, 14], NGT trees were important in maintaining the sustainability of this ancient torreya system. First, NGT trees provided rootstocks for the propagation GT trees. Seeds produced by NGT-female trees were often used to grow seedlings, most of which were used as rootstocks in the propagation of GT trees (Fig. 4a). Genetic analysis showed that no isolated pattern among different age groups of rootstocks and NGT trees (Fig. 4b), indirectly indicating that NGT saplings have been the historical source of GT rootstocks.

Second, NGT trees are important in maintaining the genetic diversity of the torreya tree system. The sign, Wilcoxon, and mode-shift tests showed that both NGT trees and GT-rootstocks did not suffer a genetic bottleneck and maintained a considerable effective population size (Additional file 1: Tables S4, S5). The microsatellite analysis indicated high genetic diversity indices in NGT trees of different age groups (Additional file 1: Table S3). Microsatellite also analysis showed that high genetic diversity was found in the rootstocks of every age group, in which GT-rootstocks were obtained from NGT trees (Fig. 3D, Additional file 1: Table S3). These results from GT rootstocks and NGT trees suggest that a high genetic diversity has been maintained in NGT trees in this ancient torreya tree system.

Why NGT tree has maintained high genetic diversity? One possible explanation is gene flow. Gene flow among populations is important in shaping and maintaining genetic diversity [15, 21, 22]. Without gene flow among populations, genetic diversity is likely to decline [23]. In our study, the gene flow analysis indicated a strong gene flow among NGT trees accrossing the six sites (Fig. 5a, b). For natural plant populations, gene flow largely results from the movement of pollen and seed [24, 25]. In agricultural systems, farmer activities (e.g., farmer-to-farmer exchanges of seed or pollen) are the important sources of gene flow [5, 26, 27]. In our study, although the local farmers are not aware that they are playing an important role in driving gene flow among populations of the species *Torreya grandis* (Additional file 1: Table S6), their practices that exchanging pollen or obtaining pollen from neighboring villages help to ensure high yields of GT seeds (Fig. 2A). Many farmers also had exchanged NGT seeds in order to produce saplings, or exchanged saplings when they propagated GT trees via grafting (Fig. 5c). These farmer activities of exchanging NGT pollens and seeds positive correlated with contemporary and historical gene flow (Table 1), suggesting that farmers that share sources of pollens and rootstocks by exchanging could promote gene flow in both short and long run within the area. Thus, the farming practices that are rooted in the accumulation of experience and knowledge by local traditional farmers would contribute to maintain high genetic diversity of this ancient torreya tree system.

Another implication NGT tree has maintained high genetic diversity might be that NGTs did not undergo a selection by farmers or modern plant breeding which might reduce effective population size [28]. Population structure and genetic diversity of crops in agriculture system usually are influenced by natural selection and human selection [29–31]. Strong selection for a special trait of crops would cause a genetic bottleneck and effective population size reduction [32]. In our study area, farmers directly used the male trees of the wild NGT as pollen providers, and used NGT saplings as rootstock for GT propagation. Our historical data also indicated NGT trees did not undergo genetic bottleneck and effective population reduction. This evidence together with farmer activities of NGT pollen and seed exchanges suggested that farmers’ practices had important role in maintaining high genetic diversity in NGT trees in this study area.

Overall, our results indicate how local farmers obtain stable seed yields, and maintain high genetic diversity of the torreya system by ingeniously using the local resources. These results enhance our understanding of how traditional agricultural systems have provided food and at the same time conserved agrobiodiversity, and how local farmers have used agrobiodiversity to maintain their agricultural systems. These results also enhance our understanding of how modern agriculture can achieve sustainability. In the development of modern agriculture, we might have more chance to meet challenge of producing more food with limited resources if we can apply the knowledge behind these traditional systems.

## Conclusion

Our results suggest that the farmers use the two types of local torreya trees (grafted and non-grafted) have helped the ancient torreya tree system to continue for thousands of years. First, the good quality and productive genotype (grafted torreya, GT), which has only female trees with genetically uniform scions and genetically diverse rootstocks, is preserved and clonally propagated via grafting. Second, the small numbers of non-grafted torreya trees (NGT trees with males and females) help maintain this ancient Chinese torreya tree system in that the male NGT trees provide pollen for both GT trees and female NGT trees, and the female NGT trees provide the seeds used to produce the saplings that are the main source of GT rootstocks. Third, gene flow among NGT trees correlated with exchange rates of pollens and saplings by farmer activities, which may contribute to the maintenance of high genetic diversity in this agriculture heritage system.

## Methods

### Study site and torreya population

Our study was conducted in the pilot site of the GIAHS-ancient Chinese torreya tree system. This pilot site, which was selected by the FAO and the United Nations Development Program (UNDP) in 2013, is located in Zhejiang Province in southeastern China (29°25′–29°47′, 120°17′–120°38′; Additional file 1: Fig. S1). The area around the site is hilly and mountainous with an altitude of about 500 m. The climate is subtropical monsoon with a mean annual air temperature of 13–15 °C and a mean annual precipitation of 1300–1560 mm. The area covers 402 km^2^ and includes three counties, 12 towns, 59 villages, and 6.8 × 10^4^ people. The characteristics of traditional farmers in this area (e.g. age, number of men and women, level of education) were shown in Additional file 1: Table S6.

The ancient torreya tree system in this part of China was begun by local farmers more than 2000 years ago [8]. It contains about 1.05 × 10^5^ ancient Chinese Torreya trees. Among them, 7.2 × 10^4^ are more than 100 years old, and thousands of them are more than 1000 years old. There are two types of torreya trees. One type is a grafted torreya (GT) tree that consists of a scion (a human-selected cultivar of *Torreya grandis* cv. *merrillii*) and a rootstock (the wild torreya of *Torreya grandis*). The other is the wild-type tree of *Torreya grandis* that is not grafted (NGT) and that is planted with GT. Local farmers have been grafting the indicated scion onto the indicated rootstocks for thousands of years [8].

### Survey of the abundance, sex ratio, and age structure of torreya trees

The field survey was authorized by the local government of Shaoxing City, Zhejiang Province, China. In the collection of plant materials, we comply with the Convention on the Trade in Endangered Species of Wild Fauna and Flora (https://www.cites.org/).

The abundance, sex ratio, and age structure of GT and NGT trees were determined by designating 47 plots (50 m × 50 m) in the six sites in the main GIAHS area (Fig. 1a). Morphological difference of GT and NGT trees was distinguished by identifying the graft scar just above ground. In each plot, we measured the location, basal diameter (BD), and sex of each of each torreya tree. The sex ratio of NGT trees was calculated in each plot. The population density (number per ha) of GT and NGT trees was statistically compared with a *t*-test in R [33].

### Age grouping based on basal diameter

Diameter at breast height (DBH) or basal diameter (BD) is often used to estimate the age of trees, especially of ancient trees [34]. Because the GT trees do not have a clear DBH, we used basal diameter (BD) to estimate the age; this is reasonable because the DBH of NGT trees is highly correlated with the BD of NGT trees (R = 0.982) (Additional file 1: Fig. S3). In estimating the age of an ancient tree, researchers usually set the initial age at 100 years [35]. In our study, we assumed that a BD of 25 cm indicated a 100-year-old tree, and four BD groups were classified as follows: (1) 25 cm < BD ≤ 65 cm (I), 65 cm < BD ≤ 105 cm (II), 105 cm < BD ≤ 145 cm (III), and 145 cm < BD ≤ 185 cm (IV). Trees were assumed to be 100–400, 400–700, 700–1000, and 1000–1300 years old in group I, II, II, and IV, respectively (Additional file 1: Table S1). In each plot, we first measured BD of each GT and NGT tree, and then assigned them to groups based on BD.

### Flowering period and duration

In sites A, B, and C, we monitored the flowering period of female and male trees of GT and NGT in each plot (Fig. 1a). The flowering period of each torreya tree was recorded every day. We classified the whole flowering period of an individual tree into three stages: initial flowering stage (10–30% of the flowers open), high flowering stage (30–100% of the flowers open), and flowering completed stage (no released pollen was observed when the tree was shaken). The flowering period of individual trees was the number of days from the initial flowering stage to the flowering completed stage. The flowering duration in a plot was the number of days from when the first tree in the plot began to flower until the last tree in the plot completed flowering.

Analysis of variance (ANOVA) was conducted in R [33]. The flowering period and duration of GT trees, NGT female trees, and NGT male trees were statistically compared with one-way ANOVA in R [33].

### Seed yield

At the six sites (Fig. 1a), the seed yields of GT and NGT trees were measured when farmers harvested. The yield was expressed as kilograms of fresh seed per tree. The temporal stability of seed yield by GT and NGT trees was compared with data from each sampling site for 6 years (2011–2016). The stability index (S) for each site was calculated as follows: S = μ/δ, where μ is the mean yield for a time period, and δ is its temporal standard deviation over the same time interval. Two-way ANOVA (type of tree and year) was performed on seed yield. The stability index (S) of GT and NGT trees were statistically compared by *t*-test after the data were square root transformed in R [33].

We also recorded the seed yield (when the farmer harvested) of each individual GT tree during 2011–2017 at site C (Fig. 1a). Each tree was assigned to one of four age groups (Additional file 1: Table S1), and yield was expressed as kilograms of fresh seed per tree. The differences in seed yield among the age groups of GT trees were assessed by one-way ANOVA in R [33].

Using the yield data from site C as described in the previous paragraph, we analyzed the stability of seed yield of each GT tree of age group III (700–1100 years old) at this site. The yield fluctuations of individual trees suggested two temporal patterns, and we divided these GT trees into two groups (A and B) according to the two patterns. The correlation of the yields of the two patterns were analyzed in R [33].

### Plant material collection and DNA extraction

At each site, both leaf and root samples were randomly collected from 20 to 70 GT trees (each individual tree as one sample, the same hereafter). Leaf samples were randomly collected from 10 to 45 NGT female trees, from 7 to 22 NGT male tree, and from 11 to 26 NGT saplings. The samples were dried and preserved in allochroic silica gel before DNA extraction.

Genomic DNA was extracted from the leaf and root samples with a DNA extraction kit (E-Zup kit, TIANGEN, China) and following the manufacturer’s protocol. All DNA samples were stored at − 80 °C for further phylogenetic and microsatellite analysis.

### cpDNA gene sequence and phylogenetic analysis

The universal primers of intergenic spacers of cpDNA were used to amplify the five sets of DNA samples (leaf and root samples of GT tree, leaf samples from female and male NGT trees, and leaf samples from NGT saplings) from three of the six sites (site A, B and C). The fragments were trnL-trnF and psbA-trnH [36]. PCR was carried out using standard protocols. The purified PCR products were sequenced by Sangon Biotech (Shanghai) Co., Ltd., China. *Torreya taxifolia* was considered as the outgroup when constructing a phylogenetic tree. The trnL-trnF and psbA-trnH sequences of cpDNA of *T. taxifolia* (GI: EF660642.1 and EF660702.1) were obtained from GenBank. The two isolated fragments were joined into one cpDNA dataset by SeqMan (DNASTAR, Lasergene 7.1). Phylogenetic relationships were determined using the maximum likelihood method (ML) performed in MEGA 6.0. Bootstrap test was performed at 1000 replicates.

### Microsatellite genotyping

Microsatellites-primers specific for *Torreya grandis* were developed by using RNA-seq. Eight leaf and root samples of *Torreya grandis* were used for RNA extraction. The cDNA library was constructed with a cDNA Synthesis Kit and sequenced on the Illumina Hiseq™ 2000 platform of Genergy Bio-tech (Shanghai, China). Each unigene was subjected to Batchprimer3 to identify potential EST-SSRs that contained at least six repeats for dinucleotides or five repeats for tri- to hexa-nucleotides. Primer pairs were designed for 5827 SSR-containing sequences based on length (18–28 bp), annealing temperatures (48–60 °C) and GC content (40–60%). The primers designed were further subjected to OLIGO v.6.67 to rule out potential mismatch, primer dimers and hairpin structures, and examined by blasting their sequences to avoid repetition. 281 EST-SSR primers were then randomly selected and synthesized for PCR amplification, and 179 of them were able to generate amplification products of expected size and band intensity in 67 individuals from three *T. grandi*s populations. Twenty primers displaying clear polymorphism among the three populations were finally selected (Additional file 1: Table S7).

The microsatellite polymorphism of DNA samples was analyzed by using 20 pairs of microsatellite primers and the following protocol. PCR reaction mixtures had a volume of 20 µL and contained 1× PCR Buffer, 2.0 Mm MgCl_2_, 0.2 mM dNTPs, primer (0.1 mM each), 1 U of Taq polymerase (TaKaRa, Japan), and 100 ng of template DNA. Cycling conditions were 95 °C for 5 min as an initial denaturation step followed by 28–37 cycles at 95 °C for 30 s, (Tm) °C for 30 s, and 72 °C for 30 s. A final extension step at 72 °C for 10 min was usually programmed as the last cycle. PCR products were separated by size with a 3730xl DNA Analyzer (Applied Biosystems, Foeter City, CA, USA) with GeneScan 500 LIZ size standard and were analyzed using GeneMarker v. 2.2.0 (SoftGenetics, Pennsylvania, USA) at Songon Biotech (Shanghai) Co., Ltd., China.

### Evaluation of genetic diversity and genetic structure

Standard measures of genetic diversity were assessed for each site based on 20 loci. These measures included genotypes, mean number of alleles per locus (Na), the effective number of alleles (Ne), observed heterozygosity (Ho), expected heterozygosity (He), and the indices of genetic diversity. The calculations were performed with GenALEx 6.502. One-way ANOVA was conducted to compare He and Na between the scions and rootstocks of GT trees, and among females, males, and saplings of NGT trees. The One-way ANOVA was also conducted to compare genotypes, He and Na among the four age groups of GT trees. All of these analyses were conducted in R [33]. A neighbor joining tree of GT trees of different age groups was constructed by using the neighbor joining method [37].

### Farmer survey of rootstock sources

We conducted a survey of how farmers obtain rootstocks for propagating GT trees at the six sites. For these interviews, we randomly selected 30–50 farmer households from 3 to 5 villages at each site (Additional file 1: Fig. S1). During the interview, we asked three questions: (i) for grafting of GT trees, did you obtain the rootstocks from seedlings that you grew, from wild seedlings, or from NGT saplings? (ii) If you grow seedlings for production of rootstocks, did you use GT seeds, local NGT seeds, or NGT seeds from other villages? and (iii) Did you grow seedlings to provide rootstocks for the local village, to provide rootstocks for the market, or to provide NGT trees for the local village.

### Analysis of genetic relationship among different age groups of the GT rootstocks and NGT trees

Using the microsatellite data, genetic distance was estimated based on Nei’s calculation [38]. Based on the genetic distance, we performed a principal coordinate analysis (PCoA) on different age groups of the rootstocks and NGT trees.

### Analysis of genetic bottleneck and effective population for NGT trees and rootstocks of GT trees

Using the microsatellite datasets of NGT female and male trees and GT rootstocks from six sites, we calculated the effective population size (Ne) based on the linkage disequilibrium method implemented in NeEstimator [39]. The minimum allele frequency cutoff was set at 0.02, and a random mating model was selected.

By using the program BOTTLENECK v 1.2.02 [40], a bottleneck was identified when observed heterozygosity (Ho) was significantly greater than expected heterozygosity (He) for a population under Hardy–Weinberg equilibrium. The computation was performed under a stepwise mutation model (SMM) and a two-phased model (TPM) in which 90% of mutations followed the SMM and 10% produced multi-step mutations; the models were assessed with sign and Wilcoxon tests. A qualitative descriptor of the allele frequency distribution (mode-shift indicator) was also used to estimate whether a population experienced a genetic bottleneck. All of these estimations were based on 1000 iterations per population.

### Evaluation of gene flow among NGT trees at different sites

Gene flow among recent generations of NGT trees in the six sites was calculated by using the model of [16]: $Nm = \frac{1 - Fst}{4Fst}$, in which N is the effective population size, m is the migration rate, and Fst is a fixation index (indicating genetic differentiation among populations). A value of Nm > 1 indicates strong gene flow.

We also estimated the historical gene flow among NGT trees at the six sites by using Migrate-n based on the Bayesian coalescent method [41, 42]. Two parameters, θ (4Neμ, where μ = mutation rate) and M (m/μ, where m = migration rate), were estimated with Migrate-n. The parameters θ and M can be used to estimate the number of migrants per generation (Nm) into each population using the model of Nm = θ × M. When Nm values > 1, there is a strong gene flow.

We ran Migrate-n with the Brownian motion microsatellite model to estimate the parameters using Bayesian inference. The initial values of θ and M were calculated by F_ST._ The uniform priors (for θ, minimum = 0.0, maximum = 0.3, delta = 0.01; for M, minimum = 0.0, maximum = 1000.0, and delta = 100.0) were then used. Four parallel chains with static heating (temperatures: 1.0, 1.5, 3.0, 100,000.0) were used. We ran each chain for 200,000 generations and sampled every 100 generations. For each chain, we used a burn-in of 20,000.

The SSR data of the four age groups (both GT and NGT trees) in three sites were used to build a neighbor joining tree using the neighbor joining method (NJ) at POPTREE 2 [37].

### Pollen and seed exchange rates, and their relationship to gene flow

We surveyed farmers in the study area concerning their exchanging of pollen to obtain high seed yield and their exchanging of NGT seeds to obtain rootstocks for the grafting of GT trees We randomly interviewed 30–50 farmer households from 3 to 5 villages at each of the six sites (Additional file 1: Fig. S1). During each interview, we provided a questionnaire to the farmers. The questionnaire asked farmers about: (i) their age, sex, education, farm size and whether they are aware of their role in this torreya system; (ii) numbers of GT and NGT trees that they planted; (iii) whether they sell or give saplings and pollen to other farmers within or outside the village; (iv) whether they buy or receive seeds and pollen from other farmers within and outside the village; (v) whether they have exchanged seeds or pollen with other farmers within their village or other villages.

We then estimated the percentage of the farmers who exchanged pollen and the total numbers of NGT seeds used within the village or traded between villages. We used this percentage to indicate the seed or sapling flow rate (exchange rate) of a village.

Mantel test [43] was conducted to evaluate the correlation between flow rates of seed and pollen and gene flow across the 6 sampling sites. Contemporary and historical gene flow rate among the corresponding site were inferred from SSR from description above.

## Supplementary information

**Additional file 1: Figure S1.** Study area, sampling site and the photos of torreya trees. **A** Map of China showing the study area. **B** Map of sampling sites (inside, the A–F indicate the 6 sample sites). **C** Grafted torreya tree. **D** Non-grafted torreya male tree. **E** Non-grafted torreya female tree. *GT* grafted torreya, *NGT* non-grafted torreya. **Figure S2**. Phylogenetic tree of *T. grandis* based on cpDNA. A, B, and C referto three sampled sites in the GIAHS area (Fig. S1). **Figure S3**. Linear relationshipbetween diameter at breast height (DBH) and basal diameter (BD) of NGT trees. **Table S1**. Relationship between basal diameter and age of a torreya tree. **Table S2**. Genetic diversity of different components in the ancient torreya tree system basedon 20 microsatellite loci. **Table S3**. Genetic diversity of GT stock and non-graftedtorreya trees in different age group. **Table S4**. The tests of effective population sizeand bottleneck for NGT-trees. **Table S5**. Effective population size and test for genetic bottlenecks in rootstocks of different ages. **Table S6**. Information of localfarmer in the study area. **Table S7**. Characteristics of 20 polymorphic microsatellite loci developed for *T. grandis*.

## Data Availability

All data generated or analyzed during this study are included in this published article and its additional information files.

## Abbreviations

GT: grafted torreya trees
NGT: non-grafted torreya trees
S: the stability index
ML: the maximum likelihood method
NJ: the neighbor joining method
Na: mean number of alleles per locus
Ne: the effective number of alleles
Ho: observed heterozygosity
He: expected heterozygosity
Nm: gene flow
