# Supplementary material for: The maintenance of stable yield and high genetic diversity in the agricultural heritage torreya tree system
Source: BMC Ecol. 2019 Sep 18;19:41. doi: 10.1186/s12898-019-0256-6 (PMC6751825; doi:10.1186/s12898-019-0256-6)
Supplement: Supplementary file 1 — Additional file 1: Figure S1. Study area, sampling site and the photos of torreya trees. A Map of China showing the study area. B Map of sampling sites (inside, the A–F indicate the 6 sample sites). C Grafted torreya tree. D Non-grafted torreya male tree. E Non-grafted torreya female tree. GT grafted torreya, NGT non-grafted torreya. Figure S2. Phylogenetic tree of T. grandis based on cpDNA. A, B, and C referto three sampled sites in the GIAHS area (Fig. S1). Figure S3. Linear relationshipbetween diameter at breast height (DBH) and basal diameter (BD) of NGT trees. Table S1. Relationship between basal diameter and age of a torreya tree. Table S2. Genetic diversity of different components in the ancient torreya tree system basedon 20 microsatellite loci. Table S3. Genetic diversity of GT stock and non-graftedtorreya trees in different age group. Table S4. The tests of effective population sizeand bottleneck for NGT-trees. Table S5. Effective population size and test for genetic bottlenecks in rootstocks of different ages. Table S6. Information of localfarmer in the study area. Table S7. Characteristics of 20 polymorphic microsatellite loci developed for T. grandis. [file 12898_2019_256_MOESM1_ESM.docx]

**The maintenance of stable yield and high genetic diversity in the agricultural heritage torreya tree system**

Jian Zhang†, Liangliang Hu†, Liang Guo, Weizheng Ren, Lufeng Zhao, Ningjing Wang, Entao Zhang, Jianjun Tang*, Xin Chen^*^

College of Life Sciences, Zhejiang University, Hangzhou 310058

* Corresponding author: [chen-tang@zju.edu.cn](mailto:chen-tang@zju.edu.cn) or chandt@zju.edu.cn

†These authors contributed equally to this paper.

**Additional Information**

**Additional Figures**

**
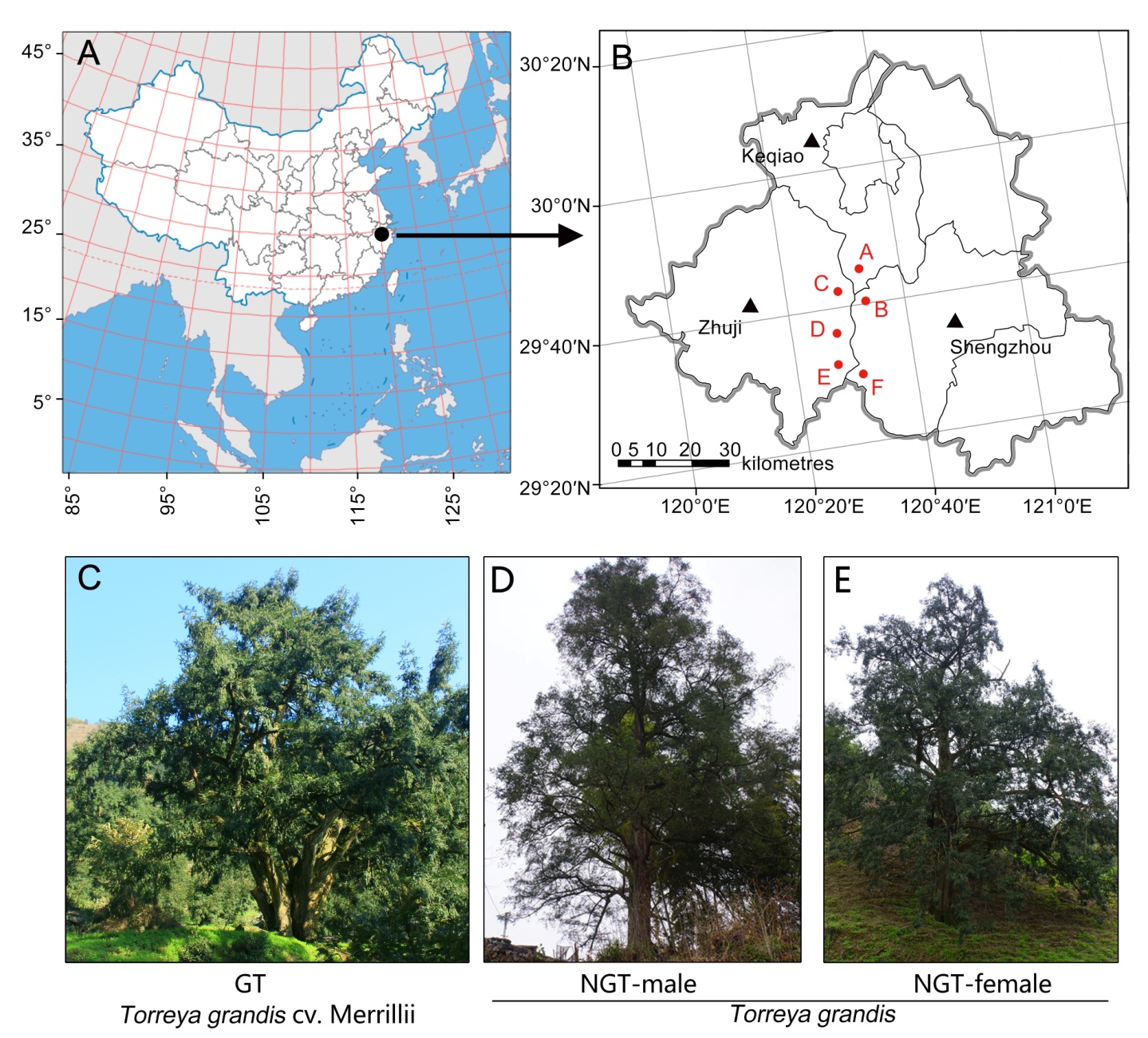
**

**Fig. S1. Study area, sampling site and the photos of torreya trees.**

A Map of China showing the study area. B Map of sampling sites (Inside, the A-F indicate the 6 sample sites). C Grafted torreya tree. D Non-grafted torreya male tree. E. Non-grafted torreya female tree. GT: grafted torreya; NGT: non-grafted torreya.

**Fig. S2.** **Phylogenetic tree of *T. grandis*** **based on cpDNA*.* A, B, and C refer to three sampled sites in the GIAHS area** (Fig. S1).

**Fig. S3. Linear relationship between diameter at breast height (DBH) and basal diameter (BD) of NGT trees.**

**Additional Tables**

**Table S1** Relationship between basal diameter and age of a torreya tree

| Age group | Class size | Basal diameter | Age |
| --- | --- | --- | --- |
| Ⅰ | ⅰ | 25cm＜BD≤45cm | 100a＜AGE≤200a |
|  | ⅱ | 45cm＜BD≤65cm | 200a＜AGE≤300a |
| Ⅱ | ⅲ | 65cm＜BD≤85cm | 300a＜AGE≤500a |
|  | ⅳ | 85cm＜BD≤105cm | 500a＜AGE≤700a |
| Ⅲ | ⅴ | 105cm＜BD≤125cm | 700a＜AGE≤900a |
|  | ⅵ | 125cm＜BD≤145cm | 900a＜AGE≤1100a |
| Ⅳ | ⅶ | 145cm＜BD≤165cm | 1100a＜AGE≤1300a |
|  | ⅷ | 165cm＜BD≤185cm | 1300a＜AGE≤1500a |

BD: basal diameter

**Table S2.** Genetic diversity of different components in the ancient torreya tree system based on 20 microsatellite loci.

| Component | N | Na | Ne | I | Ho | He |
| --- | --- | --- | --- | --- | --- | --- |
| GT-scion | 20±0 | 1.8±0.00 | 1.7±0.00 | 0.492±0.002 | 0.703±0.001 | 0.353±0.001 |
| GT-stock | 33±9 | 4.8±0.21 | 3.0±0.05 | 1.101±0.017 | 0.594±0.011 | 0.563±0.006 |
| NGT-male | 17±3 | 4.8±0.31 | 3.0±0.10 | 1.101±0.042 | 0.582±0.024 | 0.564±0.015 |
| NGT-female | 21±5 | 4.9±0.26 | 3.0±0.07 | 1.131±0.019 | 0.591±0.020 | 0.583±0.007 |
| NGT-Sapling | 16±2 | 4.3±0.28 | 2.7±0.10 | 1.029±0.034 | 0.546±0.006 | 0.541±0.010 |

N = number of individuals sampled, Na = number of alleles, Ne = effective number of alleles, I= Shannon-Weiner index, Ho=observed heterozygosity, He = expected heterozygosity. Values are means + SE. GT: grafted torreya; NGT: non-grafted torreya.

**Table S3.** Genetic diversity of GT stock and non-grafted torreya trees in different age group

| **Item** | **Age group** | **N** | **Na** | **Ne** | **I** | **Ho** | **He** |
| --- | --- | --- | --- | --- | --- | --- | --- |
| GT-stock | Ⅰ | 17±3.8 | 4.9±0.46 | 2.9±0.17 | 1.102±0.070 | 0.567±0.051 | 0.571±0.029 |
|  | Ⅱ | 14±2.6 | 4.6±0.43 | 3.0±0.19 | 1.110±0.055 | 0.603±0.038 | 0.573±0.018 |
|  | Ⅲ | 13±2.4 | 4.5±0.28 | 2.9±0.06 | 1.084±0.018 | 0.561±0.011 | 0.560±0.003 |
|  | Ⅳ | 11±2.5 | 4.2±0.37 | 2.7±0.12 | 1.049±0.034 | 0.631±0.021 | 0.556±0.003 |
| NGT-tree | Ⅰ | 19±3.2 | 5.2±0.31 | 3.1±0.06 | 1.166±0.019 | 0.571±0.018 | 0.587±0.005 |
|  | Ⅱ | 18±2.0 | 5.1±0.19 | 3.0±0.01 | 1.150±0.013 | 0.602±0.027 | 0.586±0.005 |
|  | Ⅲ | 12±0.7 | 4.7±0.24 | 3.0±0.07 | 1.128±0.035 | 0.559±0.033 | 0.575±0.013 |

Ⅰ, Ⅱ, Ⅲ, and Ⅳ refer to the four age groups based on basal diameter (Table S1). N = number of individuals sampled, Na = number of alleles, Ne = effective number of alleles, I= Shannon-Weiner index, Ho=observed heterozygosity, He = expected heterozygosity. Values are means + SE. GT: grafted torreya; NGT: non-grafted torreya.

**Table S4.** The tests of effective population size and bottleneck for NGT-trees.

| Site | Item | N | Effect population. size | |  | | Bottleneck | |  | |
| --- | --- | --- | --- | --- | --- | --- | --- | --- | --- | --- |
|  |  |  |  |  | Sign test | | Wilcoxon Test | | Mode-shift test | |
|  |  |  | 0.05 | 0.01 | TPM | SMM | TPM | SMM | | L-shaped |
| A | Male | 20 | 24.8 | 49.5 | 0.300^ns^ | 0.002* | 0.388 ^ns^ | 0.105 ^ns^ | | L-shaped |
|  | Female | 20 | 31.9 | 72.7 | 0.447 ^ns^ | 0.028* | 0.475 ^ns^ | 0.064 ^ns^ | | L-shaped |
| B | Male | 7 | 96.1 | 107.5 | 0.278 ^ns^ | 0.251 ^ns^ | 0.798 ^ns^ | 0.651 ^ns^ | | L-shaped |
|  | Female | 10 | 33.4 | 47.2 | 0.446 ^ns^ | 0.211 ^ns^ | 0.984 ^ns^ | 0.623 ^ns^ | | L-shaped |
| C | Male | 20 | 30.7 | 67.2 | 0.477 ^ns^ | 0.150 ^ns^ | 0.622 ^ns^ | 0.330 ^ns^ | | L-shaped |
|  | Female | 20 | 69.8 | 89.6 | 0.499 ^ns^ | 0.167 ^ns^ | 0.430 ^ns^ | 0.216 ^ns^ | | L-shaped |
| D | Male | 18 | 45.1 | 57.8 | 0.036* | 0.033* | 0.368 ^ns^ | 0.090 ^ns^ | | L-shaped |
|  | Female | 25 | 28.8 | 37.1 | 0.343 ^ns^ | 0.335 ^ns^ | 0.648 ^ns^ | 0.869 ^ns^ | | L-shaped |
| E | Male | 21 | 10.4 | 19.4 | 0.305 ^ns^ | 0.030 ^ns^ | 0.522 ^ns^ | 0.202 ^ns^ | | L-shaped |
|  | Female | 45 | 17.8 | 39.6 | 0.485 ^ns^ | 0.077 ^ns^ | 0.452 ^ns^ | 0.165 ^ns^ | | L-shaped |
| F | Female | 12 | 221.6 | 42.8 | 0.480 ^ns^ | 0.475 ^ns^ | 0.927 ^ns^ | 0.474 ^ns^ | | L-shaped |

A, B, C, D, E, and F refer to the six sites in the ancient torreya population, N indicates the number of samples. * indicates that the population experienced a genetic bottleneck (P＜0.05). ns indicates that the population did not experienced a genetic bottleneck (P>0.05). TPM: two-phased model; SMM: Stepwise mutation model .

**Table S5.** Effective population size and test for genetic bottlenecks in rootstocks of different ages.

| Component | Age group | N | Effect population size | |  | | | Bottleneck | | |  | |
| --- | --- | --- | --- | --- | --- | --- | --- | --- | --- | --- | --- | --- |
|  |  |  |  |  | Sign test | | | Wilcoxon test | | | Mode shaped | |
|  |  |  | 0.05 | 0.01 | TPM | SMM | | TPM | SMM | |  | |
| Rootstock | Ⅰ | 23 | 94.3 | infinite | 0.387 ^ns^ | 0.049 ^ns^ | 0.616 ^ns^ | | | 0.879 ^ns^ | | L-shaped |
|  | Ⅱ | 19 | 46.1 | 71.6 | 0.477 ^ns^ | 0.305 ^ns^ | 0.649 ^ns^ | | | 0.835 ^ns^ | | L-shaped |
|  | Ⅲ | 18 | infinite | infinite | 0.075 ^ns^ | 0.074 ^ns^ | 0.899 ^ns^ | | | 0.978 ^ns^ | | L-shaped |
|  | Ⅳ | 16 | 72.7 | infinite | 0.169 ^ns^ | 0.011* | 0.877 ^ns^ | | | 0.965 ^ns^ | | L-shaped |

Ⅰ, Ⅱ, Ⅲ, and Ⅳ refer to the four age groups based on basal diameter (Table S1). N indicates the numbers of samples; * indicates that the population experienced a genetic bottleneck (P＜0.05); ns indicates that the population did not experienced a genetic bottleneck (P>0.05).

**Table S6** Information of local farmer in the study area.

| Site | Age | Male: female | Education level(%)* | Training (%)** | Awareness (%)*** |
| --- | --- | --- | --- | --- | --- |
| A | 55 | 1:21 | 38.46 | 61.11 | 38.89 |
| B | 59 | 1:33 | 27.78 | 77.78 | 38.89 |
| C | 68 | 1:1.5 | 50.00 | 90.00 | 55.50 |
| D | 59 | 1:33 | 17.65 | 23.53 | 35.29 |
| E | 56 | 1:21 | 11.11 | 62.93 | 37.04 |
| F | 55 | 1:21 | 33.33 | 80.00 | 60.00 |

A, B, C, D, E, and F refer to the six survey sites in the ancient torreya population.

***** Education level here means percentage of farmers who receive the education of high school or above high school.

** Grafting technique training here means percentage of farmers who receive the training of grafting technique.

*** Awareness here means percentage of farmers who aware that they are playing an important role in preserving diversity of the species *Torreya grandis*,

**Table S7.** Characteristics of 20 polymorphic microsatellite loci developed for *T. grandis.*

| Locus | Primer pairs (5’-3’) | Repeat motif | Allele size range (bp) | Na | He | Ta  (℃) | GenBank accession no. |
| --- | --- | --- | --- | --- | --- | --- | --- |
| Tg1 | F:CTCTTAATAACGTGAGAACAGTG  R:ATGCTGGATAAACTCATCTTG | (AT)_7_ | 132-134 | 4 | 0.344 | 53 | MF663771 |
| Tg2 | F:TCTCAAGGCTCTCATAGATTC  R:GAGGATTCTCCTAAAGTGGAG | (GGA)_9_ | 135-138 | 3 | 0.363 | 53 | MF663772 |
| Tg3 | F:AACACGATCTTCCTTTCTCAT  R:CTCAGAGGGCTTAGTGAATTT | (AC)_7_ | 158-164 | 2 | 0.363* | 55 | MF668089 |
| Tg4 | F:TCGATCTGATAGAGAAGGTCA  R:ATTAATAAACCGCCAACACTC | (AT)_9_ | 149-171 | 10 | 0.763 | 55 | MF668090 |
| Tg5 | F:ATGATCATAAGAGGCACAAGA  R:CATTACAAAAAGGACAACAGG | (ATC)_11_ | 126-129 | 4 | 0.613 | 54 | MF668091 |
| Tg6 | F:CAGTCATTACTCCGTTTTCAC  R:TTACTACAGCTAGCCAGGTTC | (AT)_6_ | 142-153 | 7 | 0.663 | 55 | MF668092 |
| Tg7 | F:TGCTTTCAGTAGCCAGTATTT  R:CAGGGTATAGATGAGATGTGC | (TC)_12_ | 147-165 | 9 | 0.725 | 54 | MF668093 |
| Tg8 | F:GCTTGCTCTCTCTCTCTCTCT  R:TTCTTTAACCTGCCCTTTAAT | (CT)_10_ | 142-160 | 4 | 0.668 | 54 | MF668094 |
| Tg9 | F:AACCGTAGACGAGATGAGAA  R:ATGATGATATCCAGCACTGAC | (ACC)_12_ | 141-150 | 3 | 0.640 | 55 | MF668095 |
| Tg10 | F:CAATTCTTTATACGGGTTGTG  R:CGGTTGTACATTACTTTCTCTCT | (AT)_10_ | 136-168 | 20 | 0.777 | 54 | MF668096 |
| Tg11 | F:CTAGCATGTAGGGATACACGA  R:GTACGACGGACACACAATAAT | (TC)_18_ | 140-155 | 8 | 0.429 | 55 | MF668097 |
| Tg12 | F:ATGCATAGGGGACAATTTTAG  R:CTTGGTGTGGGATCTTAATG | (TG)_11_ | 144-173 | 14 | 0.801 | 55 | MF668098 |
| Tg13 | F:ATCGTCACCTTTGATTACAGA  R:AACATGGACATAAGAAGAGCA | (TCC)_6_ | 163-177 | 7 | 0.533 | 54 | MF668099 |
| Tg14 | F:GGATGGAGATGGTCTATGG  R:TCTATATAGGGAGCCGACTCTA | (AGAT)_5_ | 140-183 | 8 | 0.715 | 51 | MF668100 |
| Tg15 | F:GATGAGCATATGTGTTGTTGA  R:CAATAGAGTGTCTTCTGAGGTG | (TA)_9_ | 128-188 | 9 | 0.348 | 54 | MF668101 |
| Tg16 | F:CACTAGAACCTAACCTGCTCA  R:GTACATCAGGAATGGAATCAA | (AATC)_5_ | 141-154 | 3 | 0.629 | 54 | MF668102 |
| Tg17 | F:AACTATGCTCTCTCTCGCTCT  R:GAAAAATCCGATCTGGAATAA | (CT)_11_ | 113-131 | 12 | 0.775 | 51 | MF668103 |
| Tg18 | F:AAGCCTACCCAATTCATCTAC  R:TTTCCTACGTTCCACACTCTA | (TC)_16_ | 138-154 | 8 | 0.677 | 55 | MF668104 |
| Tg19 | F:ATGCTGTTGCTGCTTATATGT  R:GTACCATGCTTTTGATACGAA | (CGGGG)_5_ | 131-158 | 6 | 0.444 | 51 | MF668105 |
| TJ10 | F:GTCGTCACTTGTCATTCAACAA  R:TATCTTTCCCATGCCACTTC | (AT)_9_(AG)_8_ | 249-333 | 35 | 0.928 | 60 | JF754928 |

Na = number of alleles; He = expected heterozygosity.
